# Effectiveness of Companion Robot Care for Dementia: A Systematic Review and Meta-Analysis

**DOI:** 10.1093/geroni/igab013

**Published:** 2021-04-24

**Authors:** Li-Chin Lu, Shao-Huan Lan, Yen-Ping Hsieh, Long-Yau Lin, Shou-Jen Lan, Jong-Chen Chen

**Affiliations:** 1 School of Management, Putian University, China; 2 Department of Information Management, National Yunlin University of Science and Technology, Douliou, Taiwan; 3 School of Pharmaceutical Sciences and Medical Technology, Putian University, China; 4 Department of Long-Term Care, National Quemoy University, Jinning, Taiwan; 5 Department of Obstetrics and Gynecology, Chung-Shan Medical University Hospital, Taichung, Taiwan; 6 Department of Post-Baccalaureate Veterinary Medicine, Asia University, Taichung, Taiwan

**Keywords:** Agitation level, Dementia, Long-term care, Meta-analysis, Socially assistive robot

## Abstract

**Background and Objectives:**

Dementia and central nervous system degeneration are common problems in aging societies with regard to the number of people affected and total medical expenses. Socially assistive robotic technology has gradually matured; currently, most scholars believe it can be used as companions in long-term care facilities and to work as caregivers alongside staff to improve the social interaction and mental state of older adults and patients with dementia. Therefore, this study measured the effect of the duration of exposure to socially assistive robots in older adults with dementia.

**Research Design and Methods:**

Seven databases were searched up to February 2019 through the consultation of appropriate Internet sites and the use of criteria lists recommended by relevant experts. Randomized controlled trials comparing socially assistive robot use with a control group in older adults with dementia and using at least one of the primary outcomes of agitation, depression, and quality of life were included.

**Results:**

Thirteen randomized controlled trials were identified from 873 articles, 7 of which were included in the meta-analysis. The pooled effect estimate from 3 trials with 214 participants revealed that the pet-type robot improved patients’ agitation level, with a standardized mean difference of −0.37 (95% CI: −0.64 to −0.10, *p* < .01) and no heterogeneity (*I*^2^ = 0%). The results also revealed that length of each session and pet-type robot exposure time per week were associated with reduced depression levels (β = −0.06, *Q* = 21.213, *df* = 1, *p* < .001 and β = −0.019, *Q* = 7.532, *df* = 1, *p* < .01, respectively). However, the results for quality of life were nonsignificant.

**Discussion and Implications:**

Pet-type robot systems seem to be a potential activity in long-term care facilities for dementia care. Further research is warranted to establish a comprehensive intervention plan related to the use of pet-type robots.

**Translational Significance:** Pet-type robot appearance and behavioral characteristics may arouse curiosity and promote interaction in people with dementia. As a test of this effect, a pet-type robot intervention decreased depression levels according to exposure time (length of each session, total weekly exposure time). Translation efforts will need to assess the optimal length and number of sessions for this type of intervention, as well as appropriate educational training and environmental factors.

Population aging has created worldwide challenges, including increasing burden from chronic diseases such as dementia (1). The World Alzheimer Report provides a comprehensive framework for a 7-stage model for dementia services that reflects the progressive nature of dementia and subsequent care needs. Behavioral and psychological symptoms of dementia (BPSD) become more prevalent as life expectancy increases. Patients with dementia experience problems with self-care and become increasingly dependent (2). BPSD symptoms constitute mental and behavioral disturbances; psychiatric symptoms such as depression, delusions, and hallucinations; and problematic behaviors such as agitation or aggression, wandering, and apathy (3). In a sample of 555 participants from 21 centers in developing countries, 70.9% of older adults experienced at least one behavioral symptom of dementia, according to caregiver reports (4). BPSD may occur during any stage of dementia, with common symptoms including apathy (27%), depression (24%), and agitation (24%) (5). Such disturbances negatively affect prognosis, referrals, hospitalization, costs, and quality of life (QoL) in patients with dementia, and they also increase the burden on caregivers (6).

Antipsychotic medications are routinely prescribed to alleviate or eliminate BPSD in long-term care. However, research has suggested that antipsychotic drug interventions for psychiatric disorders are associated with side effects, including increased mortality rate (7,8). Psychological drugs may provide quicker and more efficient treatment of BPSD symptoms but may result in various side effects. Alternative noninvasive therapies should be identified to improve clinical symptoms and QoL for patients and caregivers. The literature describes nonpharmacological interventions (NPIs), including sensory practices, psychosocial practices, and structured care protocols. These complementary or alternative practices are safer than the use of antipsychotic drugs (9). NPI approaches focus on modifiable factors in a patient’s social and physical environment. Moreover, the benefits of NPI approaches involving tailored activities, such as music and physical activity, are unquestionable (3). NPI research of clinical or observational studies might yield benefits for BPSD symptoms. In its current state, robot technology can recognize external stimuli, collect data, apply deep learning methodologies, and conduct further analyses to generate response behavior that can achieve interaction with people; these features can be applied in dementia care for improving mental or emotional health.

Robot technologies are developing quickly. Artificial intelligence systems are designed to interact with humans and satisfy the human need for connection. Combining assistive robotics and socially interactive robotics, socially assistive robots (SARs) with audio, visual, and movement functions can assist and interact with individuals (10). Systematic reviews have reported positive (socio)psychological and physiological effects of using SARs (11–14). Moreover, SAR interventions have yielded positive effects on depression, encouraged communication, and increased social interaction (14). Social robots provide feasible alternatives for satisfying care demands; decreasing the workload of caregivers; and providing benefits such as entertainment, companionship, communication, education, and emotional support (11–13). Although these studies have provided critical information for the alleviation of BPSD symptoms; however, no standard therapy is currently available for treating dementia.

Companion robots are a subtype of SARs. They have a humanoid or animal form (most commonly they are pet-type robots) whose use in the care and well-being of older adults has been investigated (15). In recent studies, most humanoid robots were used to conduct cognitive function training for older adults or dementia patients, but the benefit was unclear till now (15). Pet-type robots have familiar animal appearances/bodies, and they, for example, exhibit active behavior and generate goals independently (16). Their use combines the advantages of animal-assisted therapy, psychological relaxation, the promotion of rehabilitation and communication, and the reduction of unpredictable clinical risks such as scratching, infection, and allergies (16).

For example, Paro can serve as a mental commitment robot that physically interacts with human beings (16,17). Paro is a seal robot with a perception system (tactile, vision, auditory, and posture sensors) (16,17). Paro has a behavior generation system that enables it to detect stimuli and respond in the form of several poses and movements, it has a reinforcement learning function (eg, it assigns the relationship between stroking and beating as positive and negative values), it reacts to sudden stimulation, and it exhibits physiological behaviors, such as diurnal rhythm (16,17). A Norwegian study involving 23 older adults with dementia engaging in group activities reported that using Paro resulted in an increase in “smiling or laughter directed at other participants” (18). In other words, the pet-type robot served not only as a “conversational partner” (19) but also as a bridge of communication with other humans (20).

However, conflicting results exist. Inconsistent findings have been obtained in previous research, in terms of agitation (21–23), depression (21,22), and QoL (21–24). In addition, researchers have not considered the role of exposure time/length of intervention. Different results have been demonstrated depending on the number of weeks of treatment: one study (22) was represented by significant differences from baseline to the end of the intervention for 12 weeks; in another study, a pooled analysis in both groups demonstrated no beneficial effects from the intervention at its endpoint (21). The application of the method (21,22) inspired us to investigate the variations in intervention time and use the change score in our meta-analysis. This study conducted a meta-analysis using intervention time to explore results, strengthen the evidence on this controversial topic, and provide clearer recommendations for the development of companion robots to achieve positive effects in dementia care.

## Method

### Data Sources and Search Strategy

The protocol for this review was accorded with the guidelines of the Preferred Reporting Items for Systematic Reviews and Meta-Analyses (PRISMA; Supplementary Table 1 and Supplementary Figure 1) (25). The PRISMA statement not only serves as a basis for reporting systematic reviews of other types of research and evaluations of interventions but also helps authors follow a clearly formulated checklist and flow diagram.

Systematic searches were conducted in February 2019 and were restricted to abstracts for peer-reviewed publications in English in the following databases: Cochrane, Embase, EBSCO host/PsycINFO, Ovid Medline, PubMed, the Web of Science, and the IEEE digital library (Xplor). Search terms were based on the intervention (ie, socially assistive robots; eg, social*, assist*, interact*, robot*) and the context (ie, elderly care; eg, old adult, older adult, old age, silver, older person, elder*, aging, and dement*, Alzheimer*, cognit*). Therefore, the search term included 2 parts. The asterisk (*) was used as a truncation symbol substituting any potential part of the word during the searches.

In addition, manual searches of related articles were conducted to obtain additional potentially eligible studies (snowball procedure). All references searched and studies for inclusion were imported into EndNote X9, and the key information extracted was independently screened by 2 reviewers.

### Inclusion and Exclusion Criteria

Included studies fulfilled the following criteria: (a) the study design was a randomized controlled trial (RCT) with comparison groups—experimental group and control group (with control conditions or no intervention); (b) study participants were older adults with cognitive impairment or a clinical diagnosis of dementia or Alzheimer disease based on a mental examination/test (such as the Mini-Mental State Examination or Clinical Dementia Rating Scale) or documented in medical records, from accredited staff members or professional care providers; (c) the interventions involved SARs, which communicated or interacted with people by using sensors in the form of verbal communication (such as for humanoid robots), nonverbal communication (such as for pet-type robots), or both; and (d) outcomes were agitation, depression, and QoL.

Studies were excluded if they fulfilled the following criteria: (a) review articles and letters to the editor; (b) papers from annual conferences, international conferences, workshops, international symposiums, progress reports, and presentations; (c) studies assessing robots’ acceptability to users; (d) articles focused on companion robots’ development and usability; and (e) studies of interventions using physically or surgical assistance robots.

### Data Extraction and Quality Assessment

For title and abstract screening and the extraction of data from studies meeting the inclusion criteria, 2 investigators independently assessed the eligibility of all studies retrieved from the databases on the basis of predetermined selection criteria. The following information was extracted: first author’s name, publication year, country, study design, intervention (related to SARs), duration of follow-ups, and outcomes of agitation, depression, and QoL. However, if quantitative data were not provided in the studies, approximate values were obtained from the figures or calculated from proportions. For example, if an included article presented outcomes on a line graph or box graph, or exact data were not provided for the change score, we adopted approximate values by using an online ruler tool (26) for a line graph or box graph or calculation of a change score (final data − baseline data).

Two investigators independently applied the Cochrane Collaboration tool for the risk of bias to assess the methodological quality of the included RCTs and evaluate the possibility of bias in the design of each included study. Studies were assessed after a description of their design contents according to the items in the Cochrane Collaboration tool. These included selection bias, performance bias, detection bias, attrition bias, and reporting bias (27). Any discrepancies in study selection were resolved through discussion.

### Statistical Analysis

Data were entered into Review Manager Software (RevMan 5.4; computer program; The Cochrane Collaboration, 2020) and Comprehensive Meta-Analysis software (version 2.0; Biostat, Englewood, NJ) to assess treatment efficacy and publication bias. In the meta-analysis of comparative studies, weighted mean differences and corresponding 95% confidence intervals (CIs) estimated for the various studies were pooled using random-effects models (DerSimonian and Laird method) (28), and pooled effect estimates were calculated. Meta-regression was used to evaluate the effect of 6 types of intervention time, namely total SAR intervention period (number of weeks), total SAR intervention sessions (number of sessions per week × number of weeks), weekly SAR intervention frequency (total number of sessions/number of weeks), total SAR exposure time (number of sessions × length of each session × number of weeks), length of each session, and SAR exposure time per week (number of weekly sessions × length of each session).

The heterogeneity of intervention effects between studies was assessed through *I*^2^ and the Cochran *Q* tests; significance for heterogeneity was set at *I*^2^ more than 50% (29). Publication bias was examined using funnel plots to assess the validity of the effect estimates versus standard error, visual assessment of a funnel plot was drawn, Begg’s test to evaluate the correlation between the ranks of effect sizes, Egger regression asymmetry test to examine the association between estimated intervention effects, and a measure of study size (27,30,31).

## Results

### Identification of Relevant Studies

**Figure 1.**
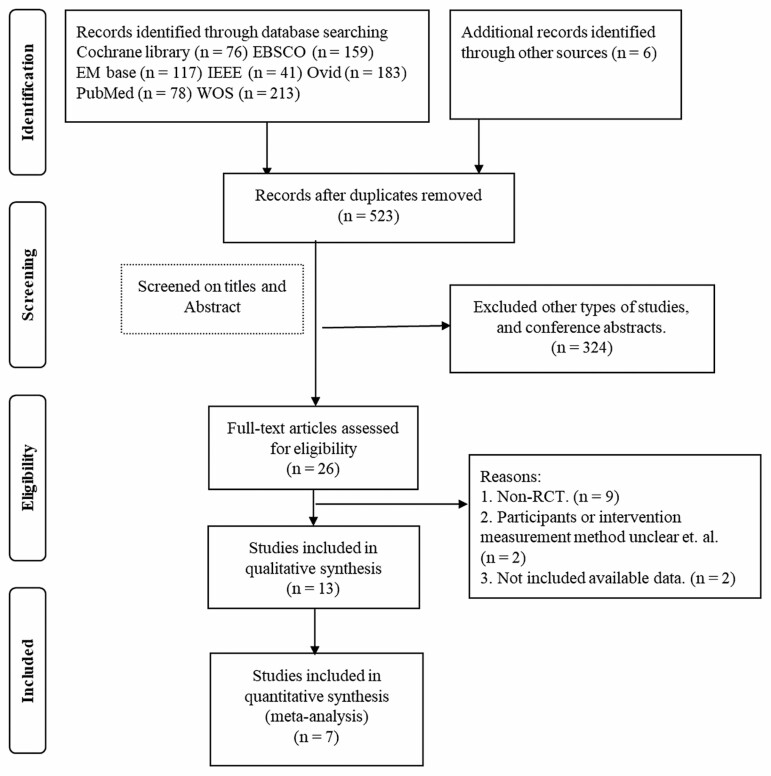
Flowchart of the study selection process. *Notes:* The electronic searches found a total of 867 records and 6 additional snowball records; of these 26 full texts were retrieved for closer examination. A total of 13 articles were included in the final review and 7 studies were included in the meta-analysis. RCT = randomized controlled trial.

The electronic database search yielded 867 records and 6 additional snowball records; in total, 324 records were screened by title and abstract and were excluded because of irrelevance to the review objectives; for example, participants were not older adults with cognitive impairment and/or with dementia and/or the intervention did not involve SAR and/or the study design did not mention a comparison group. Of these, 26 records were identified for full-text screening. Studies were excluded for the following reasons: they were not RCT, participants were not older adults with dementia/cognitive impairment, the intervention involved physical assistant robots or surgical assistant robots, intervention processes and measurements were not described clearly, and data on outcomes were unavailable; and 13 records were eligible for inclusion. Finally, 7 articles (32–38) met the inclusion criteria, with 3 articles reporting on agitation outcomes, 4 on depression, and 3 on QoL (Figure 1; Table 1).

**Table 1.** Study Characteristics of Companion Robot Intervention for Dementia Care (*n* = 13)

Study, Country	Facility	Duration of intervention	Patient/Participant Dementia Stage (Women %; M/F) Age (range; mean)	Dementia Definition	Intervention, *N*	Results Outcome (tool)	Outcome Measure Included in the Meta-analysis	Quality* a b c d e f
Moyle et al., 2013 (39), Australia	Long-term care facilities	45 min/session, 3 afternoons/week, 5 weeks	Mid- to late-stage dementia (Unclear) Mean: 85.3	DSM, fourth edition	Pet-type robot Paro = 9 Control (reading) = 9	*Increase*: quality (QoL-AD) pleasure (OERS) behaviors: wandering (AWS)		+ − − + + + High
Robinson et al., 2013 (32), New Zealand	Hospital and rest home areas	60 min/session, 2 afternoons/week, 12 weeks	Cognitive impairment: 48% (Unclear) 55–100 years	Cognitive impairment: Abbreviated Mental Test ≤6	Pet-type robot Paro = 17 Control (crafts, movies, or bingo) = 17	*No main effects*: quality (QoL-AD) Staff-rated Quality of life *Slightly decreased*: depression (GDS)^1^ loneliness (UCLA) >adjusting problem P663	Depression QoL	+ + − −+ + High
					Pet-type robot Paro = 11 Resident dog = 17	*Behavior*: touched and talked to the robot more than a dog		
Bemelmans et al., 2015 (44), The Netherlands	Psychogeriatric care	15 min/session, 5 times per month, 2 months	Dementia phase: in the first stages of dementia: most of the participants in the final stage of dementia: 6 (14/57) Unclear	The Dutch 28-item version of the GIP-28 scale	91 participants of which 71 completed the study Pet-type robot Paro = 69 Control (care support) = 17	*Decreased*: GIP-28 scale for psychogeriatric inpatients		− − −? + + Low
Joranson et al., 2015 (33), Norway	Nursing homes	30 min/session, twice per week, 5 weeks	Cognitive impairment/moderate–severe dementia (67% women) 62–95 years	Norwegian version: a. dementia diagnosis (MMSE score of 7/30), Clinical Dementia Rating Scale b. Cognitive impairment (MMSE score of 25/30)	Pet-type robot Paro = 27 Control (no intervention) = 26	*Decreased*: agitation (BARS) depression (CSDD)	Agitation Depression	+ + − − + + High
Soler et al., 2015 (34), Spain	Nursing homes	30–40 min/session, 2 days/week, 12 weeks	Moderate/severe dementia (88% women) Mean age: 84.68 (58–100 years)	Diagnosis of neurodegenerative dementia	2 year-long phases: Phase 1: Pet-type robot Paro = 33 humanoid social robot NAO = 30 Control (conventional therapy—perform several therapeutic activities) = 38	*Increase*: Deterioration (GDS)^2^ irritability/lability (NPI) *No main effects*: cognitive (SMMSE) quality (QUALID) neuropsychiatric (NPI) total score *Decreased*: apathy (APADEM-NH)	QoL	+? −? + + Low
					Phase 2: Pet-type robot Paro = 42 Dog = 36 Control (conventional therapy—perform several therapeutic activities) = 32	Deterioration (GDS) quality (QUALID) neuropsychiatric (NPI)		
Joranson et al., 2016 (35), Norway	Nursing homes	30 min/session, twice per week, 12 weeks	Cognitive impairment/mild/moderate/severe dementia (67% women) 62–95 years	Norwegian version: a. Dementia diagnosis (MMSE score of 7/30), Clinical Dementia Rating Scale b. Cognitive impairment (MMSE score of 25/30)	Pet-type robot Paro = 27 Control (usual care) = 26	*Increase*: quality (QoL-AD) for severe dementia *Decreased*: medication use	QoL	+ − − − + + Low
Thodberg et al., 2016 (42), Denmark	Nursing home	Unclear 12 biweekly visits, 9 am–4 pm 6 weeks	Mild/moderate/severe dementia (69% women) 79–90 years Not all residents were diagnosed, even though they showed clear signs of having some form of dementia	MMSE	Pet-type robot Paro = 35 Living dog = 35 Toy cat = 30	*Increase (third week)*: Sleep duration *No main effects (sixth week)*: cognitive (MMSE) depression (GDS) *Decreased (after)*: depression (GDS)		+? − ? +? Low
Thodberg et al., 2016 (43), Denmark	Nursing home	Unclear 12 biweekly visits, 9 am–4 pm 6 weeks	Mild/moderate/severe dementia (69% women) 79–90 years Not all residents were diagnosed, even though they showed clear signs of having some form of dementia	MMSE	Pet-type robot Paro = 35 Living dog = 35 Toy cat = 30	*Increase*: interaction—physical contact, eye contact verbal communication		+ + − ? + + High
Liang et al., 2017 (36), New Zealand	Dementia day care centers and at home	30 min/session, 11 am–12 pm 2 to 3 times a week 6 weeks	Dementia_unclear (64% women) 67–98 years	Formal diagnosis of dementia	*Day care* Pet-type robot Paro = 13 Control (standard care) = 11 *Home setting* Pet-type robot Paro = 13 Control (standard care) = 11	*Increase*: interaction (facial expression, talked to staff) depression (CSDD) *No main effects*: agitation (CMAI-SF) neuropsychiatric (NPI-Q) total score	Home setting: Agitation Depression	+ + − − + + High
Moyle et al., 2017 (37), Australia	Long-term care facilities	15 min/session, 3 afternoons/week, 10 weeks	Dementia_unclear (76% women) Mean: 85	A documented diagnosis of dementia	Pet-type robot Paro = 138 Plush toy = 140 Control (usual care_music, physical activity, and mental stimulation) = 137	*Improving*: agitation (CMAI-SF) iMood states (video observation) Increase: engagement-verbally, visually engaged	Agitation	+ + − + + + High
Petersen et al., 2017 (38), USA	Dementia units	20 min/session, 3 times/week, 12 weeks	Mild to moderate dementia (77% women) Mean: 83.4	DSM	Pet-type robot Paro = 35 Control (standard care) = 26	*Increase*: anxiety (RAID) depression (CSDD) pulse oximetry *Decreased*: pulse rate medication utilization	Depression	+ + − ?? + Low
Jones et al., 2018 (40), Australia	Long-term care facilities	15 min/session, 3 afternoons/week, 10 weeks	Various types of dementia (73% women) Mean: 84	Had a diagnosis of dementia	Pet-type robot Paro = 138 Control (unclear)	*Greater positive*: behavioral engagement, visual engagement [assessed by video observation] *Fewer*: observed instances of agitation (CMAI-SF)		+? −? + + Low
Moyle et al., 2018 (41), Australia	Long-term care facilities	15 min/session, 3 afternoons/week, 10 weeks	Various types of dementia (76% women) Mean: 85	Had a diagnosis of dementia and documented in medical and care records	Pet-type robot Paro = 67 Plush toy = 55 Control = 53 Nighttime: Pet-type robot Paro = 98 Plush toy = 95 Control (usual care) = 87	*Greater reduction*: “in daytime step count, in nighttime step count, daytime physical activity measured by SenseWear armbands”		+ + − + + + High

*Notes:* F = female; M = male; DSM = Diagnostic and Statistical Manual; MMSE = Mini-Mental State Examination; NAO = a humanoid robot; OERS = Observed Emotion Rating Scale; Paro = a pet robot; sMMSE = Severe Mini Mental State Examination; QoL-AD = Quality of Life for Alzheimer’s Disease; AWS = Revised Algase Wandering Scale—Nursing Home version; GDS^1^ = Geriatric Depression Scale; GDS^2^ = the Global Deterioration Scale; UCLA = UCLA Loneliness scale; BARS = Brief Agitation Rating Scale; CSDD = Cornell Scale for Symptoms of Depression in Dementia; NPI = Neuropsychiatric Inventory; QUALID = Quality of Life in Late-stage Dementia; APADEM-NH = Apathy Scale for Institutionalized Patients with Dementia Nursing Home version; CMAI-SF = Cohen–Mansfield Agitation Inventory—Short Form; NPI-Q = Neuropsychiatric Inventory Brief Questionnaire Form; RAID = Rating for Anxiety in Dementia; GIP-28 = Behavior Rating Scale for psychogeriatric inpatients.

*Quality of bias assessment with Cochrane Collaboration Tool (a = selection bias, b = selection bias, c = detection bias, d = attrition bias, e = reporting bias, f = other bias) and all enrolled studies were confirmed as being high quality (a score of >3); +, low-risk bias; −, high-risk bias; ?, unclear.

### Study Characteristics and Study Quality

**Figure 2.**
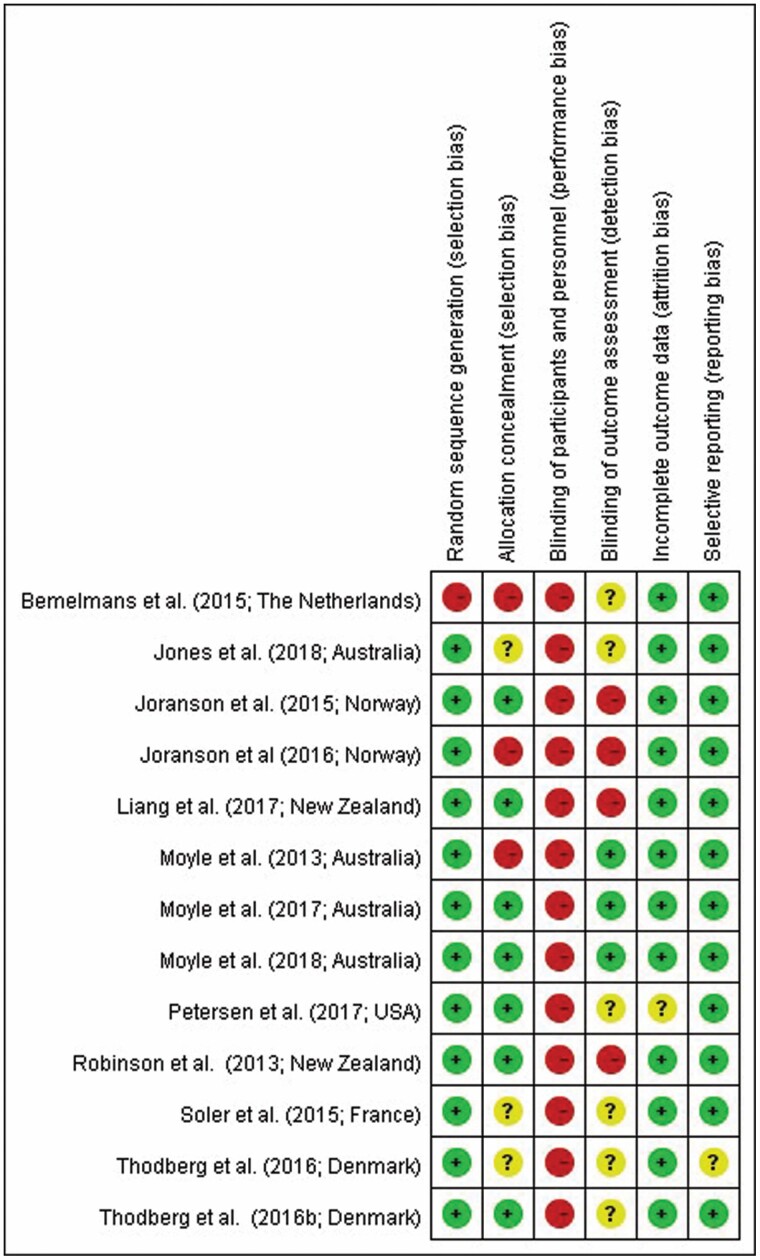
Risk of bias summary. *Notes*: Assessment of risk of bias of the included articles revealed in the risk of bias summary which question mark presented unclear, positive mark presented low risk of bias, negative mark presented high risk of bias.

Between 2013 and 2019, 13 studies were published in Western countries: 4 were from Australia (37,39–41), 2 were from Norway (33,35), 2 were from New Zealand (32,36), 2 were from Denmark (42,43), 1 was from the United States (38), 1 was from the Netherlands (44), and 1 was from Spain (34). Thirteen studies recruited older people with dementia. Most of the participants were women; all of the studies’ participants lived in long-term care facilities, including 2 dementia units, 1 psychogeriatric care unit, and 1 hospital and rest home areas (Table 1). In this article, 6 studies were considered to be of high quality, others were low quality. The risk of bias summary for each RCT study, including selection, performance, attrition, detection, and reporting bias, according to the Cochrane Collaboration’s items and guidelines, is outlined in Figure 2. Quality scores ranged from 0 to 6, and all enrolled studies were confirmed as being high quality (a score of >3), more than half of the total score, after discussions among our team members and assess the reliability statistical power of the 13 articles (Cronbach alpha 0.935).

### Effectiveness Outcomes

#### Agitation

The 3 studies included in the agitation analysis indicated that participants with dementia who underwent a pet-type robot intervention exhibited significant changes in agitation. Three studies assessed agitation levels, with 105 participants in the robot intervention group and 109 in the control group (33,36,37). The pooled results indicated a significant decrease in agitation levels, with a standardized mean difference (SMD) of −0.37 (95% CI: −0.64 to −0.10, *p <* .01) and without heterogeneity (*I*^2^ = 0%; Table 2; forest plot presented in Supplementary Figure 2).

**Table 2.** Forest Plot of Agitation, Depression, and Quality of Life

Outcome or Subgroup	Studies	Participants	Statistical Method	Effect Estimate
Agitation	3	214	Std mean difference (IV, fixed, 95% CI)	−0.37 [−0.64 to −0.10]**
12 W	1	24	Std mean difference (IV, fixed, 95% CI)	0.08 [−0.72 to 0.89]
15 W	1	139	Std mean difference (IV, fixed, 95% CI)	−0.46 [−0.80 to −0.13]**
24 W	1	51	Std mean difference (IV, fixed, 95% CI)	−0.32 [−0.88 to 0.23]
Depression	4	170	Std mean difference (IV, random, 95% CI)	−0.10 [−0.52 to 0.31]
QoL	3	161	Std mean difference (IV, fixed, 95% CI)	0.17 [−0.15 to 0.48]

*Note:* IV = iinverse variance; QoL = quality of life, CI = confidence interval.

*p < .05, **p < .01.

#### Depression

The participants with dementia who underwent the pet-type robot intervention exhibited no significant changes in depression between the baseline and at 12 weeks. Four studies assessed depression, with 90 participants in the robot intervention group and 80 in the control group (32,33,36,38). The pooled results indicated no significant differences in depression, with an SMD of 1.22 (95% CI: −0.79 to 3.22, *p* = .23) and with heterogeneity (*I*^2^ = 97%; Table 2; forest plot presented in Supplementary Figure 2).

#### Quality of life

Three studies assessed QoL, with 86 participants in the pet-type robot intervention group and 75 in the control group (32,34,35). The pooled results indicated no significant difference in QoL, with an SMD of 0.13 (95% CI: −0.41 to 0.67, *p* = .63) and with heterogeneity (*I*^2^ = 64%; Table 2; forest plot presented in Supplementary Figure 2).

### Publication Bias

The outcomes of funnel plots (presented in Supplementary Figure 3), Begg’s test, and Egger’s test were used to evaluate the publication bias of meta-analyses for at least 3 studies. Agitation outcomes, Egger’s test and Begg’s test indicated no significant publication bias (*p* = .08 and .14, respectively); depression outcomes, Egger’s test indicated no presence of bias (*p* = .08), whereas Begg’s test revealed substantial publication bias (*p* = .04); and QoL outcomes indicated no publication bias (Egger’s test *p* = .33, Begg’s test *p* = .50).

### Meta-regression

**Figure 3.**
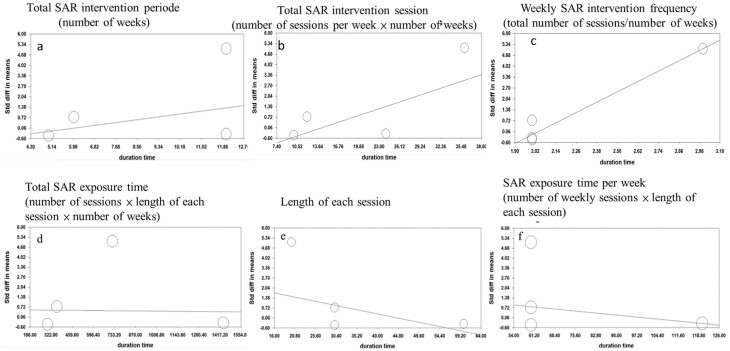
Meta-regression of depression of 6 types of intervention time. The meta-regression results revealed that exposure time could reduce depression level. Six types of intervention time on depression: (**A**) total SAR intervention time (number of weeks): β = 0.213, *Q* = 14.861, *df* = 1, *p* < .001; (**B**) total SAR intervention sessions (number of sessions × number of weeks): β = 0.137, *Q* = 46.305, *df* = 1, *p* < .001; (**C**) weekly SAR intervention frequency (number of sessions [frequency/week]): β = 5.210, *Q* = 85.923, *df* = 1, *p* < .001; (**D**) total SAR exposure time (number of sessions × length of each session × number of weeks): β = −0.00009, *Q* = 0.064, *df* = 1, *p* = .800; (**E**) length of each session: β = −0.06, *Q* = 21.213, *df* = 1, *p* < .001; (**F**) SAR exposure time per week (number of sessions × length of each session): β = −0.019, *Q* = 7.532, *df* = 1, *p* = .006. SAR = socially assistive robot.

A quantitative analysis of depression was used to analyze the effect of the intervention, including the 6 types of intervention time. Meta-regression was used to analyze depression outcomes after the companion robot intervention according to the 6 types of intervention time. The results revealed that 2 time factors had a positive effect on depression levels: length of each session (β = −0.06, *Q* = 21.213, *df* = 1, *p* < .001; Figure 3E) and total pet-type robot exposure time each week (β = −0.019, *Q* = 7.532, *df* = 1, *p* < .01; Figure 3F). However, 3 types of exposure frequency had a negative effect on depression; weekly exposure frequency (β = 5.210, *Q* = 85.923, *df* = 1, *p* < .001; Figure 3C) had a greater negative effect than total number of weeks and total frequency of interventions (β = 0.213, *Q* = 14.861, *df* = 1, *p* < .001 and β = 0.137, *Q* = 46.305, *df* = 1, *p* < .001, respectively; Figure 3A and B; Supplementary Table 2).

## Discussion

The notable findings of this systematic review and meta-analysis study are that pet-type robot care demonstrates benefits for the relief of BPSD in patients with dementia. With respect to BPSD specifically, the results demonstrate significant reductions in agitation. Moreover, pet-type robot interventions varied by the number or length of each pet-type robot session and the number of weeks for overall exposure. The meta-regression results indicated that exposure time (length of each session and total exposure time each week) of the pet-type robot interventions improved depression over time. For QoL, no significant effects were identified. These results may serve as inspiration for other studies to develop individualized stimulation programs; pet-type robot care might be useful in group treatment processes or even as a method for facilitating interaction between patients with dementia and their caregivers and therapists.

### Agitation

Our results are consistent with those of studies indicating that pet-type robots can alleviate patients’ agitation (21,22), but inconsistent with those of Pu et al. (23). Despite no statistically significant results being revealed in the previous meta-analysis (23), positive effects were observed on neuropsychiatric symptoms, including decreased agitation. When the results for the pet-type robot groups in the 3 studies were considered individually, the postintervention agitation level was noted to be reduced (33,36,37). Notably, Liang et al. (36) found that both experimental and control groups experienced improved agitation, implying that the home care setting might be a factor influencing agitation level.

Three studies used Paro as an intervention, with the staff members having already taken a mandatory course on how to conduct the intervention sessions (33,36,37). For the intervention, an indoor space was used as the activity space, the group was small (within 6 attendees in a group (33,36)), the intervention frequency was at least twice a week, and a total pet-type robot exposure time of 45–60 minutes per week was applied (33,36,37). These intervention elements might therefore be conducive to building interaction opportunities or allowing relaxation. These findings imply that before introducing pet-type robots, staff member training, environment setting, and activity time arrangement can be used to improve the outcomes.

### Depression

Our results are consistent with those of studies reporting that pet-type robots had a nonsignificant impact on depression (21,23) but inconsistent with those of Leng et al. (22). Each study provided detailed information on pooling the results of depression data, and the included articles differed according to the study’s research objectives and selection criteria. For example, Pu et al. (23) evaluated the effectiveness of social robots for older adults and included 7 studies not specifically designed for older adults with dementia. Similarly, another 2 studies also included studies according to their research objectives to conduct data analysis (21,22); therefore, it is inappropriate to compare the results related to depression among those studies.

Although studies have reviewed the impact of pet-type robots on depression, none had analyzed the effect of the intervention. This study provides the first results regarding the intervention time (stratified into 6 types) with pet-type robots on older adults with dementia. We divided the temporal intervention data into intervention frequency and exposure time per session. Various aspects of intervention frequency, including the total intervention period with a pet-type robot, the total number of sessions, and weekly frequency, were assessed through direct meta-regression. A negative effect on depression was observed in older adults with dementia, which may be attributed to overly frequent exposure and a lack of novelty (eg, 2 or 3 sessions a week).

By contrast, exposure to the pet-type robot ameliorated depression, especially a 30- to 60-minute-long session (32,33) and weekly exposure of 60–120 minutes (32,33). However, these findings should be interpreted with caution because only 2 articles were considered to derive these findings. According to Wada et al. (16), Paro was created as a mental commitment robot, which is defined as an artificial emotional creature that provides physical interaction, varied stimulation, and playful interaction; these might help interpret changes in emotional activity. In everyday communication, people may express their thoughts and emotions only after spending a certain amount of time with their friends/trusted people. Future studies should further explore the effect of the duration of exposure on participants interacting with pet-type robots.

### Quality of Life

The pooled results of this study are consistent with studies reporting that pet-type robot interventions revealed no difference in QoL compared with the control group (21–23). Several studies to date have contributed knowledge regarding robotic biofeedback (36,38,45) and identified the potential effectiveness of SARs for reducing BPSD by ameliorating agitation and depression (22) and positively affecting QoL (24); one review (46) used 5 distinct SARs (Paro, NAO, CRECA, Betty, and Haptic Creature) to investigate the potential of this technology to affect mental health outcomes (comfort or companionship and stress reduction) among older residents in nursing home facilities. However, QoL should be interpreted with caution because this study pooled results of only RCTs using Paro (*n* = 3).

### Challenges

In general, 2 major aspects should be considered before a pet-type robot intervention is implemented (Supplementary Table 3). (a) The patients’ disease severity at baseline (including low-to-moderate severity of agitation) (40) as well as preferences and willingness to interact with pet-type robots (33,34) must be known to develop care plans and increase patient participation. In addition, brain dysfunction caused by pathological changes, such as β-amyloid deposition, might affect attentional control (or distractibility); patients with dementia may exhibit a lack of attentional control (47). Caregivers and family members require education regarding the progressive nature of dementia and how it affects an individual’s overall function, such as inattention and BPSD. In particular, care targets promote understanding among participants’ family members, enabling them to more effectively manage patients with dementia (36,37). (b) Care procedures, including dementia care education training, are necessary, and the intervention duration must be considered along with the intervention environment. Trained staff members should follow the same introduction script in each session to minimize the risk of confusion (33–37,39–41,44). Those setting intervention durations must consider the condition of patients with dementia patients to suit their daily rhythm (42,43).

In summary, the main point is educational training, including basic knowledge of patients (34,35,38,40) and their willingness to use SARs (34,36,42,43), observation and listening skills related to dementia (44) and care-related abilities (32,34,40,41), explanation of the activity specifications (33,34,36,39), leading the residents to interact (32,33,36,38,39), and respecting participants’ right to decide how they interact (37). Another point is environmental factors, which include having a dedicated barrier-free space for residents (33,34,39,44) and participants forming a circle for activities (33–35,38). Future research could examine the aforementioned ideas using a rigorous study design and applying a pet-type robot intervention.

### Limitations

The present systematic review and meta-analysis were subject to several limitations: (a) “assistive social robots” are not consistently and clearly defined in articles on robots. This study used interactive autonomous robots, including artificial intelligence systems, social commitment robots, mental commitment robots, companion robots, and assistive robots. However, articles on the use of robots for rehabilitation and therapy were not included in this study. (b) Some research designs were not robust, described the intervention process in insufficient detail, and used inadequate sample sizes. The limited sample size of less than 30 should also be taken into account during the interpretation of such results. (c) Heterogeneity existed among the studies in the meta-analysis, perhaps as a result of differences among participants, executors, institutions, and intervention processes. (d) No precision tools have assessed the effects of SARs on (socio)psychological and physiological outcomes. More research is required to confirm the effect of SARs on (socio)psychological and physiological outcomes. (e) Cultural factors and the use of different tools to diagnose dementia mean that dementia has different definitions. In addition, differences in dementia awareness, care, and health care services caused differences in the intervention process. (f) Finally, this systematic review and meta-analysis included only a small number of studies, so our results should be interpreted with caution.

## Conclusions

Pet-type robots could stimulate interaction, alleviates agitation, and have positive effects on depression in patients with dementia. They look like play the role of companion. Pet-type robot systems seem to be a potential activity in long-term care facilities for dementia care. Additional research is required to experimentally investigate the effects of the duration of exposure featuring pet-type robotics within a variety of older people health care settings.

In addition, educational training (eg, having appropriate knowledge of patients’ hobbies and respecting participants’ rights) and environmental factors (eg, paying attention to the influence of the physical environment and strengthening observation and listening skills) must be considered before the initiation of pet-type robot interventions. Further research is urgently required in the field of pet-type robots (AIBO, NeCoro, and Haptic Creature), and a comprehensive patient-centered plan must be designed for pet-type robot therapy programs.

## Supplementary Material

igab013_suppl_Supplementary_Materials
