# Supplementary material for: Effectiveness of Companion Robot Care for Dementia: A Systematic Review and Meta-Analysis
Source: Innov Aging. 2021 Apr 24;5(2):igab013. doi: 10.1093/geroni/igab013 (PMC8304164; doi:10.1093/geroni/igab013)
Supplement: igab013_suppl_Supplementary_Materials [file igab013_suppl_supplementary_materials.docx]

**Online Supplementary Material for Publication in *Innovation in Aging*:**

**Effectiveness of Companion Robot Care for Dementia: A Systematic Review and Meta-Analysis**

Li-Chin Lu, PhD^1,2^, Shao-Huan Lan, PhD^3^, Yen-Ping Hsieh, PhD^4^, Long-Yau Lin, MD, DSc^5^, Shou-Jen Lan, DDS, MPH, PhD^6^, Jong-Chen Chen, PhD^2,*^

1. School of Management, Putian University, China

2. Department of Information Management, National Yunlin University of Science and Technology, Taiwan

3. School of Pharmaceutical Sciences and Medical Technology, Putian University, China

4. Department of Long Term Care, National Quemoy University, Taiwan

5. Department of Obstetrics and Gynecology, Chung-Shan Medical University Hospital, Taiwan

6. Department of Post-Baccalaureate Veterinary Medicine, Asia University, Taiwan

*Address correspondence to: Jong-Chen Chen, PhD, Department of Information Management, National Yunlin University of Science and Technology, 123 University Rd. Douliu, Taiwan, R.O.C. E-mail: [jcchen@yuntech.edu.tw](mailto:jcchen@yuntech.edu.tw)

**Supplementary Table 1. PRISMA Checklist**

| **Section/topic** | **#** | **Checklist item** | **Reported on page #** |
| --- | --- | --- | --- |
| TITLE | | |  |
| Title | 1 | Identify the report as a systematic review, meta-analysis, or both. | P1 |
| ABSTRACT | | |  |
| Structured summary | 2 | Provide a structured summary including, as applicable: background; objectives; data sources; study eligibility criteria, participants, and interventions; study appraisal and synthesis methods; results; limitations; conclusions and implications of key findings; systematic review registration number. | Abstract |
| INTRODUCTION | | |  |
| Rationale | 3 | Describe the rationale for the review in the context of what is already known. | P3-5 |
| Objectives | 4 | Provide an explicit statement of questions being addressed with reference to participants, interventions, comparisons, outcomes, and study design (PICOS). | P5-6 |
| METHODS | | |  |
| Protocol and registration | 5 | Indicate if a review protocol exists, if and where it can be accessed (e.g., Web address), and, if available, provide registration information including registration number. | none |
| Eligibility criteria | 6 | Specify study characteristics (e.g., PICOS, length of follow-up) and report characteristics (e.g., years considered, language, publication status) used as criteria for eligibility, giving rationale. | P7-8 |
| Information sources | 7 | Describe all information sources (e.g., databases with dates of coverage, contact with study authors to identify additional studies) in the search and date last searched. | P7 |
| Search | 8 | Present full electronic search strategy for at least one database, including any limits used, such that it could be repeated. | P7 |
| Study selection | 9 | State the process for selecting studies (i.e., screening, eligibility, included in systematic review, and, if applicable, included in the meta-analysis). | P7-8 |
| Data collection process | 10 | Describe method of data extraction from reports (e.g., piloted forms, independently, in duplicate) and any processes for obtaining and confirming data from investigators. | P9 |
| Data items | 11 | List and define all variables for which data were sought (e.g., PICOS, funding sources) and any assumptions and simplifications made. | P9 |
| Risk of bias in individual studies | 12 | Describe methods used for assessing risk of bias of individual studies (including specification of whether this was done at the study or outcome level), and how this information is to be used in any data synthesis. | P9 |
| Summary measures | 13 | State the principal summary measures (e.g., risk ratio, difference in means). | P8-10 |
| Synthesis of results | 14 | Describe the methods of handling data and combining results of studies, if done, including measures of consistency (e.g., I^2^) for each meta-analysis. | P8-10 |
| Risk of bias across studies | 15 | Specify any assessment of risk of bias that may affect the cumulative evidence (e.g., publication bias, selective reporting within studies). | P9-10 |
| Additional analyses | 16 | Describe methods of additional analyses (e.g., sensitivity or subgroup analyses, meta-regression), if done, indicating which were pre-specified. | P9-10 |
| RESULTS | | |  |
| Study selection | 17 | Give numbers of studies screened, assessed for eligibility, and included in the review, with reasons for exclusions at each stage, ideally with a flow diagram. | P11 |
| Study characteristics | 18 | For each study, present characteristics for which data were extracted (e.g., study size, PICOS, follow-up period) and provide the citations. | P11 |
| Risk of bias within studies | 19 | Present data on risk of bias of each study and, if available, any outcome level assessment (see item 12). | P11-12 |
| Results of individual studies | 20 | For all outcomes considered (benefits or harms), present, for each study: (a) simple summary data for each intervention group (b) effect estimates and confidence intervals, ideally with a forest plot. | P12 |
| Synthesis of results | 21 | Present results of each meta-analysis done, including confidence intervals and measures of consistency. | P13 |
| Risk of bias across studies | 22 | Present results of any assessment of risk of bias across studies (see Item 15). | P12-13 |
| Additional analysis | 23 | Give results of additional analyses, if done (e.g., sensitivity or subgroup analyses, meta-regression [see Item 16]). | P13 |
| DISCUSSION | | |  |
| Summary of evidence | 24 | Summarize the main findings including the strength of evidence for each main outcome; consider their relevance to key groups (e.g., healthcare providers, users, and policy makers). | P14-16 |
| Limitations | 25 | Discuss limitations at study and outcome level (e.g., risk of bias), and at review-level (e.g., incomplete retrieval of identified research, reporting bias). | P17-18 |
| Conclusions | 26 | Provide a general interpretation of the results in the context of other evidence, and implications for future research. | P18-19 |
| FUNDING | | |  |
| Funding | 27 | Describe sources of funding for the systematic review and other support (e.g., supply of data); role of funders for the systematic review. | P19 |

*From:*  Moher D, Liberati A, Tetzlaff J, Altman DG, The PRISMA Group (2009). Preferred Reporting Items for Systematic Reviews and Meta-Analyses: The PRISMA Statement. PLoS Med 6(6): e1000097. doi:10.1371/journal.pmed1000097

For more information, visit: **www.prisma-statement.org**.

**Supplementary Figure 1 PRISMA-2009-Flow-Diagram.**


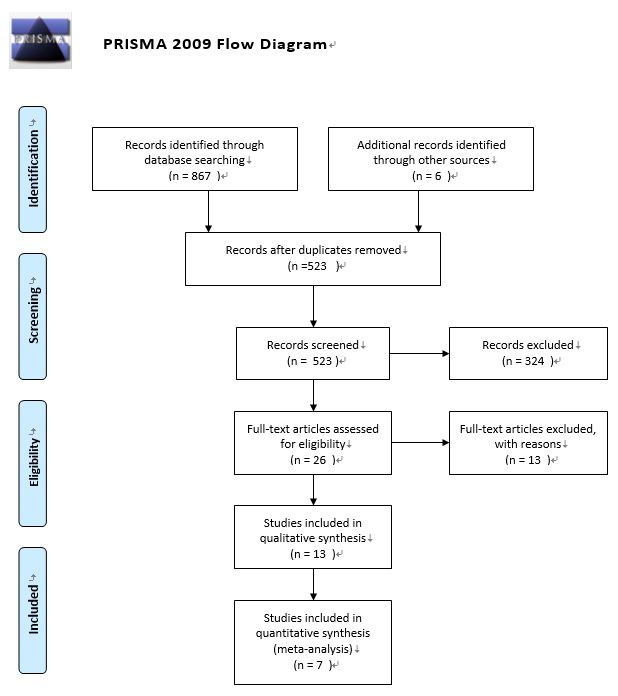


*From:*  Moher D, Liberati A, Tetzlaff J, Altman DG, The PRISMA Group (2009). *P*referred *R*eporting *I*tems for *S*ystematic Reviews and *M*eta-*A*nalyses: The PRISMA Statement. PLoS Med 6(6): e1000097. doi:10.1371/journal.pmed1000097

**For more information, visit** [**www.prisma-statement.org**](http://www.consort-statement.org/)**.**

**Supplementary Figure 2. Forest plot of Agitation (A) and Depression (B) and Quality of life (C).**


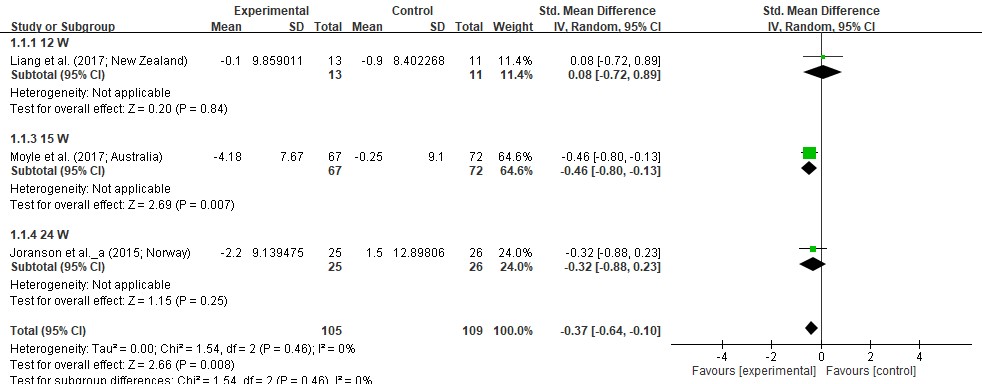


1. **Agitation**


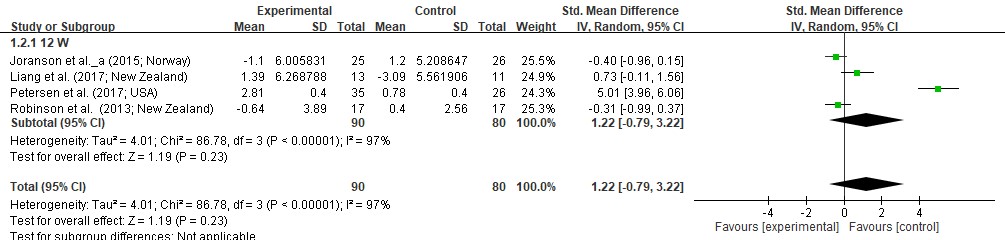


1. **Depression**


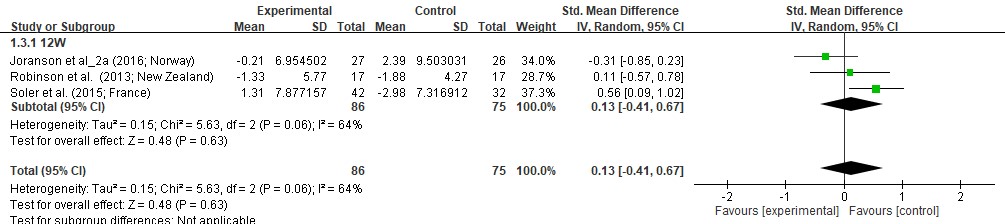


1. **Quality of life**

**Supplementary Figure 3. Funnel plot of Agitation (A) and Depression (B) and Quality of life (C).**


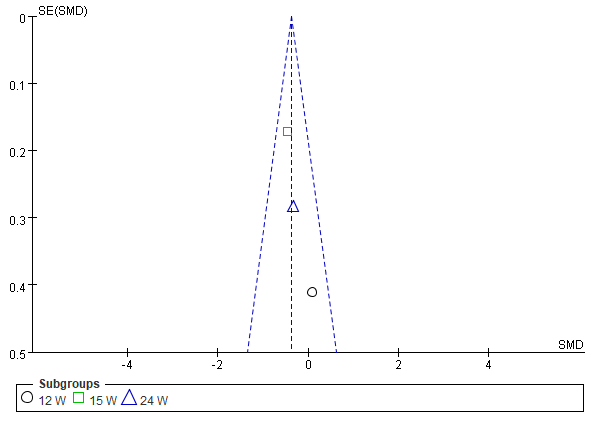


1. **Agitation**


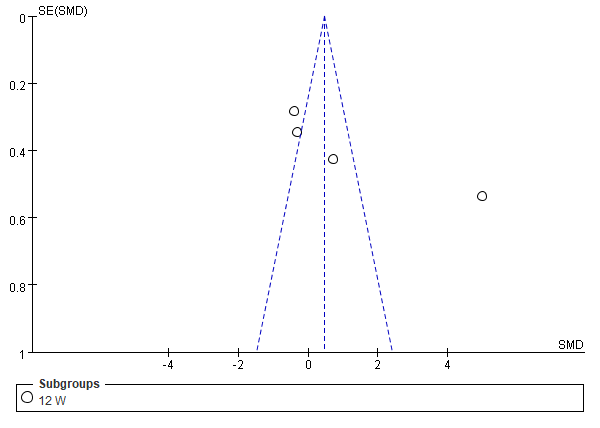


1. **Depression**


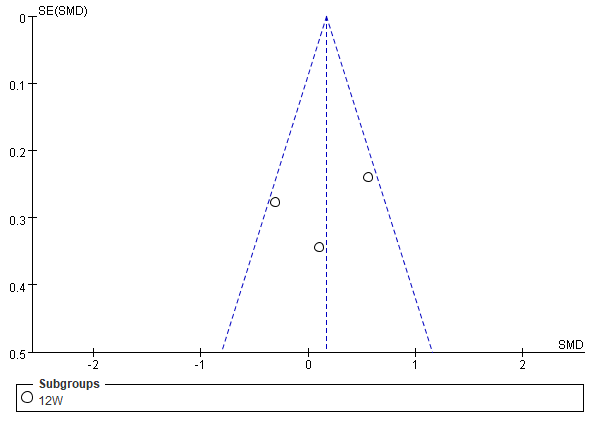


1. **Quality of life**

**Supplementary Table 2. Six types intervention time on depression by meta-regression**

| **Frequency** | |
| --- | --- |
| **Total SAR intervention periode:** number of weeks | (β = 0.213, Q = 14.861, df = 1, P < .001***) |
| **Total SAR intervention session:** number of sessions per week × number of weeks | (β = 0.137, Q = 46.305, df = 1, P < .001***) |
| **Weekly SAR intervention frequency:** total number of sessions/number of weeks | (β = 5.210, Q = 85.923, df = 1, P = < .001***) |
| **Time** | |
| **Total SAR exposure time:** number of sessions × length of each session × number of weeks | (β = -0.00009, Q = 0.064, df = 1, P = .800) |
| **Length of each session** | (β = -0.06, Q = 21.213, df = 1, P = < .001***) |
| **SAR exposure time per week:** number of weekly sessions × length of each session | (β = -0.019, Q = 7.532, df = 1, P = .006**) |
| Note:  Depression data retrieval from GDS or CSDD by secondary data, high score is mean heig depression. Change value (post-pre), negative values represent improved. GDS: Geriatric Depression Scale CSDD: Cornell Scale for Depression in Dementia p < .05*, p < .01**, p < .001*** | |

**Supplementary Table 3. Study of socially assistive robots (SARs) instructions for dementia care.**

| Study, country | Facility | Indoor or outdoor  Companion robot intervention | Lessons learned |
| --- | --- | --- | --- |
| Moyle et al,^39^ 2013, Australia | Long-term care facilities | **Indoor (Staffs receive training for practicing)** 1. Using the concepts of discovery, attracting an emotional response, social interaction with each other through **discussion** and touching Paro. 2. the facilitator showed Paro to each individual and demonstrated how Paro responded. | 1. Positioning of the intervention within the facility. 2. The dedicated space had no barriers, such as doors (encouraged some participants to walk away from the intervention group) 3. Paro is used with individuals or in small groups of up to three residents (Nine participants with one Paro would be not recommend) 4. Suggest Paro activity requires facilitation rather than Paro being left with a resident. |
| Robinson et al,^32^ 2013,  New Zealand | Hospital and rest home areas | **Indoor** discussion groups were held and all residents had a chance to interact with the robot. | During normal activities: 1. It was noted how often residents talked to each other  2. How the presence of the seal robot or the resident dog altered the social atmosphere 3. Providing a conversation topic for residents and staff |
| Bemelmans et al,^44^ 2015, The Netherlands | Psychogeriatric care Dementia | **Indoor (Staffs receive training for practicing)** making Paro interventions part of the daily care routine, which providing comfort to individual distressed dementia, or aims at facilitating the provision of daily care tasks by care staff. | 1. An additional load on the caregivers. 2. Taking into account the possible learning curve when applying Paro in a care-support activity 3. Should be paid to hygiene |
| Joranson et al,^33^ 2015, Norway | Nursing homes | **Indoor (Staffs receive training for practicing)** 1. Take place in a separate, quiet room. 2. All participants sit close together in a half circle without a table in front of them, and sit in their usual seats (up to 6 participants in a group). 3.Each session started with a presentation of Paro as an articulated toy to reduce misinterpretations. 4. Activities naturally occurring between each participant and Paro, such as petting, talking to and about, smiling to, and singing for equal periods of time. | Residents showed an interest in Paro during recruitment would contribute to willingness join the activity. |
| Soler et al,^34^ 2015, Spain | Nursing homes | **Indoor (Staffs receive training for practicing)** 1. Patients with mild and moderate dementia were in a group and individual sessions were used with patients with moderate-severe and severe dementia.  2. The group sessions were conducted with 9–15 participants seated in a circle. 3. All sessions had the same overall structure: greeting the group, introduction, therapeutic exercises (cognitive or physical therapy) and ending. | 1. Criteria were not fear of the robot or dog and non-severe acute illness. 2. The regular therapists performed the sessions. 3. To pay close attention to the dementia demands and their associated environmental impact implications. |
| Joranson et al,^35^ 2016, Norway | Nursing homes | The same with Joranson et al. (2015), Norway | The differential severity of the dementia through with Paro intervention change in quality of life. |
| Thodberg et al,^42^ 2016a, Denmark | Nursing home | **Indoor (Staffs receive training for practicing)** 1. Each visit lasted 10 min. (Each participant received a total of 12 visits) 2. The ‘animals’ had to be within reach of the resident for at least 80% of the time, unless the resident clearly showed no interest at all or disapproved of contact with the ‘animal’. | 1. The visits was chosen to suit the daily rhythm of the individual participant. 2. The effect of different intervention durations has not yet been studied. |
| Thodberg et al,^43^ 2016b, Denmark | Nursing home | The same with Thodberg et al. (2016), Denmark | Some residents were very fascinated by the robot, others did not find it interesting (its novelty value declines over time.) |
| Liang et al,^36^ 2017, New Zealand | Dementia day care centers and  at home | **Indoor (Staff and caregivers receive training for practicing)** Day care sites: 1.1100 and 1200 hours at day care sites, 2 to 3 sessions each week for 6 weeks (the sessions lasted for 90 min). 2.in a separate room with 3 to 6 attendees.  Home: 1. each dyad for 6 weeks. 2.It suggested letting the relative hold and stroke Paro, talking with the relative about Paro. | Day care sites: each participant for up to 5 minutes with Paro such as stroking Paro’s flippers, to encourage care recipient interactions. Home: the length and nature of interactions with Paro at home was flexible and depended on each dyad’s needs and preferences.  It is therefore important to understand that Paro’s therapeutic benefit depends on individual users’ needs and desires. |
| Moyle et al,^37^ 2017, Australia | Long-term care facilities | **Indoor (Staffs receive training for practicing)** 1. RAs left the participant with the PARO to interact with it as they liked, returning after 15 minutes to collect PARO.  2. All sessions were conducted during the afternoon hours of1:00 PM-5:00 PM (when agitation levels are commonly highest) | Participants to decide how they would interact and use PARO. |
| Petersen et al, 2017,^38^ USA | Dementia units | **Indoor (Staffs receive training for practicing)** 1. The 20-minute sessions involved seating 6 residents at a round table.  2. Placing the PARO robotic pet in the center of the table, and encouraging the residents to interact with the robotic pet by demonstrating interaction | Knowledge regarding robotic biofeedback that can reduce the amount of antidepressant medication. |
| Jones et al,^40^ 2018, Australia | Long-term care facilities | The same with Moyle et al. (2017; Australia) | Participants with more severe agitation at baseline had a poor response to PARO, with the level of baseline agitation being the key factor. |
| Moyle et al,^41^ 2018, Australia | Long-term care facilities | The same with Moyle et al. (2017; Australia) | Non-facilitated sessions with PARO had some effect in reducing motor activity (include wandering, restlessness, rocking and repetitious mannerism) when compared to a plush toy comparison and usual facility care. |

*Note*. PARO (pet-type robot)
